# Early Laparoscopic Cholecystectomy for Acute Cholecystitis. When Do Risks Seem Imminent?

**DOI:** 10.1111/ases.70052

**Published:** 2025-05-06

**Authors:** Salah Mansor, Amine Zaidi, Mohammad Habibullah, Rizeq Hourani, Yazan Aldali, Mohamed Said Ghali, Salahaldeen Dawdi, Idress Suliman, Mohammed Alobahi, Lutfi Jarboa, Mohamed Valiyapurayil, Ahmad Zarour

**Affiliations:** ^1^ Acute Care Surgery Section Hamad General Hospital, HMC Doha Qatar; ^2^ General Surgery Department Al‐Jalla Teaching Hospital, Benghazi University Benghazi Libya; ^3^ College of Medicine Qatar University Doha Qatar; ^4^ General Surgery Department Ain Shams University Cairo Egypt; ^5^ Weill Cornell Medical College Doha Qatar

**Keywords:** acute cholecystitis, delayed cholecystectomy, early cholecystectomy, laparoscopic cholecystectomy

## Abstract

**Introduction:**

Surgery for acute cholecystitis is time‐critical; the timing of laparoscopic cholecystectomy in acute cholecystitis patients has historically been controversial because of a perceived increased risk of complications. The aim is to evaluate the impact of operative timing within 7 days of symptom onset on patient outcomes.

**Method:**

A retrospective cohort study of patients who underwent laparoscopic cholecystectomy within 7 days after being admitted for acute cholecystitis between January 2016 and December 2021 in the Acute Care Surgery section. The study was conducted by dividing the study population into seven groups based on the operation day for each patient to evaluate the impact of operative timing on postoperative outcomes and compare the clinical results to determine how long the operation will be safe.

**Results:**

Within the study period, 3299 acute cholecystitis patients underwent laparoscopic cholecystectomy. The mean age was 42.4 years, with 50.1% of them being women and 49.9% of them being men. The rate of patients older than 65 years was 6.2%. A total of 237 patients (7.18%) had complications; the conversion to open surgery occurred in 27 patients (0.8%); and the overall reoperation rate was 0.5% (17 patients).

**Conclusion:**

Our study shows that delays in laparoscopic cholecystectomy scheduling for acute cholecystitis after 3 days from the onset of symptoms can lead to a longer operative duration as well as a longer hospital stay. However, it does not significantly impact overall complications and reoperation rates, allowing a feasible and safe procedure to be performed within 7 days.

## 1 Introduction

Gallbladder diseases are considered the most common pathology of the biliary tree and the common reason for surgical department admissions [1, 2]. In the Western world, approximately 15% of the population has asymptomatic gallstones, whereas up to 25% of them may experience biliary colic, with 4% of these cases progressing to acute cholecystitis or biliary pancreatitis [3]. The most frequent reason for acute cholecystitis is stones obstructing the gallbladder outflow, whereas in 5%–10% of cases, it is caused by gallbladder stasis, which leads to a local inflammatory reaction in the gallbladder wall [4, 5].

Surgical intervention remains the curative method for symptomatic gallbladder stones. For the last several decades, the gold standard treatment of acute cholecystitis has been achieved through laparoscopic cholecystectomy: This operation is considered the commonest abdominal surgical procedure performed in daily elective as well as urgent surgery lists worldwide [1, 6]. However, the optimal timing for performing laparoscopic cholecystectomy remained a subject of controversy among surgeons for an extended period. In the last century, there were two approaches to dealing with acute cholecystitis: Early surgery versus initial conservative treatment followed by delayed laparoscopic cholecystectomy several weeks later, which is recognized as early laparoscopic cholecystectomy, performed within 7 days from the onset of symptoms, and delayed laparoscopic cholecystectomy, performed within 6 weeks to 3 months [7]. Cochrane review comparing early with delayed surgery found no difference in complication rates while showing a shorter hospital stay in the early group [8]. Several international studies have shown that up to 30% of people scheduled for delayed laparoscopic cholecystectomy are readmitted with gallstone‐related complications before their operation [9]; in the United Kingdom, there were more than 130 000 admissions with gallstone complications in 1 year due to prolonged waiting time for elective cholecystectomy [10].

In a study to determine the most optimal time for early laparoscopic cholecystectomy, it was found that those who underwent laparoscopic cholecystectomy within 72 h of admission had a shorter operative duration and a shorter hospital stay compared with those who underwent laparoscopic cholecystectomy between the 4th and 7th day [11]. However, it is important to mention that aside from hospital stays and duration of the operation, there was no significant difference among patients who underwent laparoscopic cholecystectomy during the 4th, 5th, 6th, and 7th days of admission, indicating that up to 7 days, because admission can be safely done [11]. Some international studies have concluded that early operations within 72 h had an advantage in terms of shorter operation time and hospital stays without a significant increase in the open conversion rate in comparison to late operations that were done three to 6 weeks later [12]. More studies were done on whether timing should be initiated from the onset of symptoms or admission. Two meta‐analyses showed that timing is based on the onset of symptoms, with cholecystectomy being performed within 72 h of symptoms found to significantly reduce postoperative complications compared with delayed laparoscopic cholecystectomy [13, 14]. The 2018 Tokyo Guidelines recommended early laparoscopic cholecystectomy in low‐risk patients with acute cholecystitis regardless of the amount of time that has passed since symptom onset and concluded that there are still benefits to performing laparoscopic cholecystectomy in patients for whom more than 72 h had passed since symptom onset [15]. According to the World Society of Emergency Surgery 2020 guidelines, early laparoscopic cholecystectomy is superior to delayed laparoscopic cholecystectomy for acute cholecystitis as it results in a significantly lower complication rate, a lower length of hospital stays, and an earlier return to work [7].

Despite the advances in acute care and surgical techniques, there is still significant morbidity and mortality associated with acute cholecystitis and its surgical treatment. Therefore, this study aims to evaluate the impact of the timing of the operation on patients by comparing the clinical outcomes of individuals who underwent laparoscopic cholecystectomy each day within 7 days of symptom onset to determine how long the operation will be safe and when the risk is looming on the horizon.

## 2 Methods

In a retrospective cohort study of consecutive, nonselected patients who underwent laparoscopic cholecystectomy within 7 days after being admitted for acute cholecystitis between January 2016 and December 2021 in the Acute Care Surgery section, to assess the effect of operative timing on postoperative outcomes, the study population was divided into seven groups according to the day of each patient's operation, where the safety of operative timing was determined by comparing the clinical outcomes of each day within the first 7 days of symptom onset. Table 1 provides the basic characteristics of the study population, whereas Table 2 shows the summary of patient demographic characteristics of the study population.

**TABLE 1:** Summary of the patient basic characteristics of the study population.

Age	> 65 years	< 65 years	*p*
1 day	30 (5.5%)	513 (94.5)	0.512
2 days	72 (6.2%)	1083 (93.8%)	
3 days	44 (6.3%)	649 (93.7%)	
4 days	24 (6%)	377 (94%)	
5 days	18 (9.2%)	178 (90.8%)	
6 days	9 (6.9%)	122 (93.1%)	
7 days	7 (3.9%)	173 (96.1%)	
Gender	Male	Female	*p*
1 day	277 (51%)	266 (49%)	0.486
2 days	566 (49%)	589 (51%)	
3 days	348 (50.2%)	345 (49.8%)	
4 days	209 (52.1%)	192 (47.9%)	
5 days	105 (53.6%)	91 (46.4%)	
6 days	69 (52.7%)	62 (47.3%)	
7 days	79 (43.9%)	101 (56.1%)	
Body mass index	Mean	SD	*p*
1 day	28.98	6.01	0.188
2 days	29.25	8.68	
3 days	29.53	13.28	
4 days	29.61	11.07	
5 days	29.87	9.82	
6 days	31.48	22.56	
7 days	29.8	7.05	
Past biliary history	Yes	No	*p*
1 day	291 (53.6%)	252 (46.4%)	0.001
2 days	675 (58.4%)	480 (41.6%)	
3 days	408 (58.9%)	285 (41.1%)	
4 days	194 (48.4%)	207 (51.6%)	
5 days	92 (46.9%)	104 (53.1%)	
6 days	56 (42.7%)	75 (57.3%)	
7 days	100 (55.6%)	80 (44.4%)	
Diabetic mellitus	Yes	No	*p*
1 day	81 (14.9%)	462 (85.1%)	0.039
2 days	154 (13.3%)	1001 (86.7%)	
3 days	110 (15.9%)	583 (84.1%)	
4 days	77 (19.2%)	324 (80.8%)	
5 days	28 (14.3%)	168 (85.7%)	
6 days	29 (22.1%)	102 (77.9%)	
7 days	27 (15.0%)	153 (85.0%)	
Previous abdominal surgery	Yes	No	*p*
1 day	124 (22.8%)	419 (77.2%)	0.701
2 days	276 (23.9%)	879 (76.1%)	
3 days	150 (21.6%)	543 (78.4%)	
4 days	81 (20.2%)	320 (79.8%)	
5 days	48 (24.5%)	148 (75.5%)	
6 days	26 (19.8%)	105 (80.2%)	
7 days	42 (23.3%)	138 (76.7%)	

**TABLE 2:** Summary of the patient demographic characteristics of the study population.

No.	Variable		*N*	%	Mean ± SD
1	Sex				
	Male		1646	49.9%	
	Female		1653	50.1%	
2	Mean age				42.42 ± 12.7 years
	> 65		204	6.2%	
	< 65		3095	93.8%	
3	BMI				29.47 ± 10.6 kg
4	Past biliary history		1816	55%	
5	DM		506	15.3%	
6	History of another comorbidity		1283	38.9%	
7	Past abdominal surgery		747	22.6%	
8	History of bariatric surgery		255	7.7%	
9	Postoperative complication		237	7.2%	
10	Duration from pain onset to the operation day	Day 1	543	16.5%	
		Day 2	1155	35%	
		Day 3	693	21%	
		Day 4	401	12.2%	
		Day 5	196	5.9%	
		Day 6	131	4%	
		Day 7	180	5.5%	

The diagnosis of acute cholecystitis was made by the guidelines outlined in the Tokyo guidelines. Intraoperative findings and histopathology reports after surgery confirmed the diagnosis. Perioperative data include age, gender, body mass index, past medical history, diabetes mellitus, past biliary history, previous abdominal and bariatric surgery, onset of symptoms, time of the operation, duration of operation, conversion to open surgery, and complications including intraoperative, biliary, and postoperative complications, the reoperation rate, and the length of hospital stays. All this data was collected. The primary endpoint was the appearance of complications, whereas the secondary endpoint was passing 6 weeks of follow‐up after discharge. To assess the distribution of data in statistical analysis, the mean ± standard deviation (SD) was used for all continuous variables, whereas frequency and percentage were used for categorical data. When appropriate, the Fisher exact test for categorical variables or the *X* ^2^ test were used to compare groups. *p* values of less than 0.05 were regarded as statistically significant in the statistical analyses carried out with the SPSS v21 statistical program.

The study was reviewed and approved by the ethics committee (IRB number: MRC‐01‐24‐236); in addition, informed consent was obtained as the hospital is a teaching hospital.

## 3 Results

**FIGURE 1:**
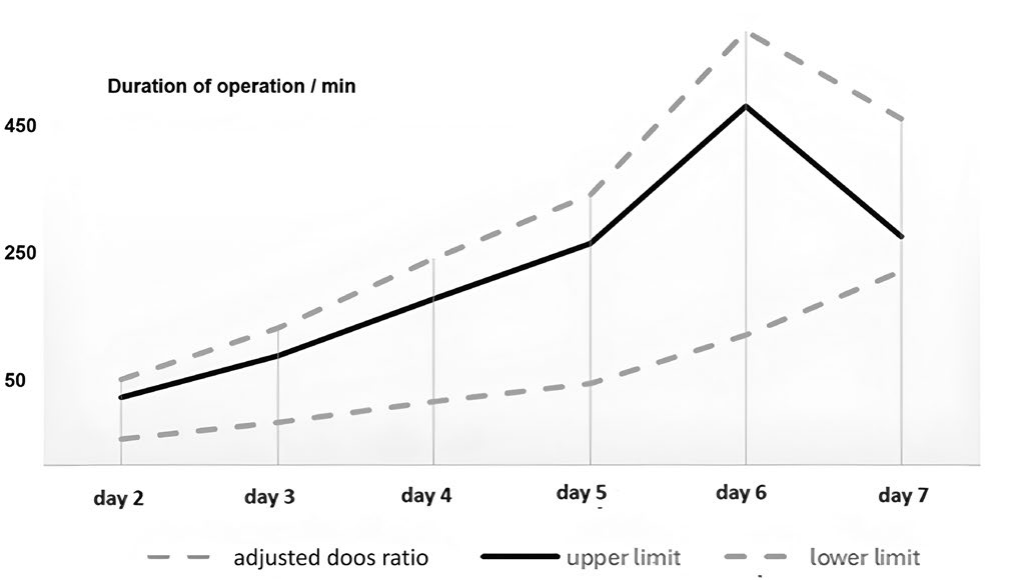
Changing the operation duration according to the timing of surgery.

Within the study period, 3299 acute cholecystitis patients underwent laparoscopic cholecystectomy. The mean age of the patients was 42.42 + 12.7 years (range: 15–99), with 50.1% of them being women and 49.9% of them being men. Patients over 65 made up 6.2% of the total. The baseline characteristics in Table 1 across the groups indicated statistically significant differences only in past biliary history, suggesting that those with a history of biliary disease are more likely to develop complications than those without. Table 2 summarizes the study population's fundamental demographic features and distribution by operation day. Concerning the relation between the duration of the operation and the date of the procedure, as shown in Figure 1, patients had a significantly longer operative duration if the surgery was delayed from admission day versus 7 days after admission (*p* < 0.001).

**FIGURE 2:**
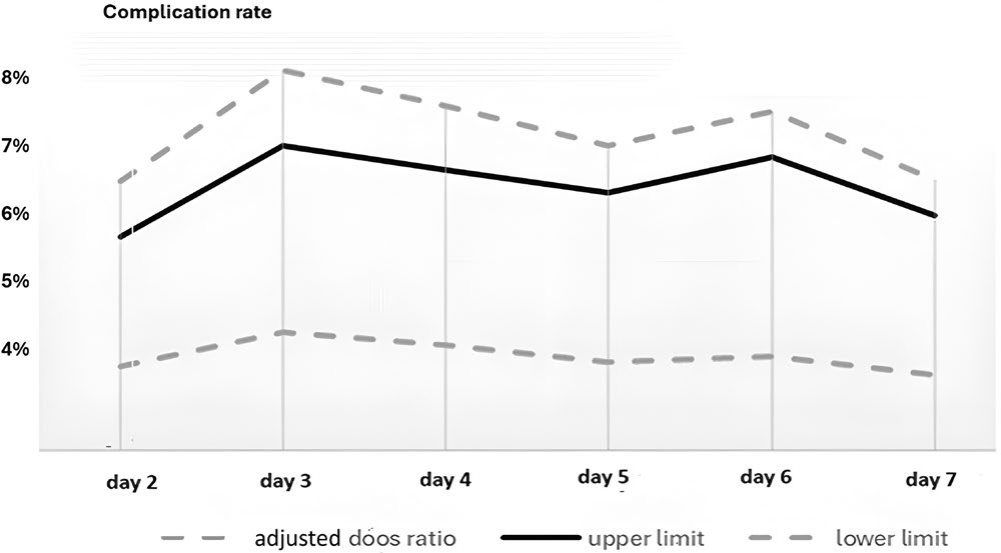
The rate of complications every day within the first week.

The complications in the study cases accounted for 237 patients (7.18%); Table S1 provides the details of postoperative complications of the study population. As shown in Figure 2 there was no significant difference in the number of total complications for patients who underwent laparoscopic cholecystectomy on the first day and subsequently within 7 days (*p* = 0.373).

**FIGURE 3:**
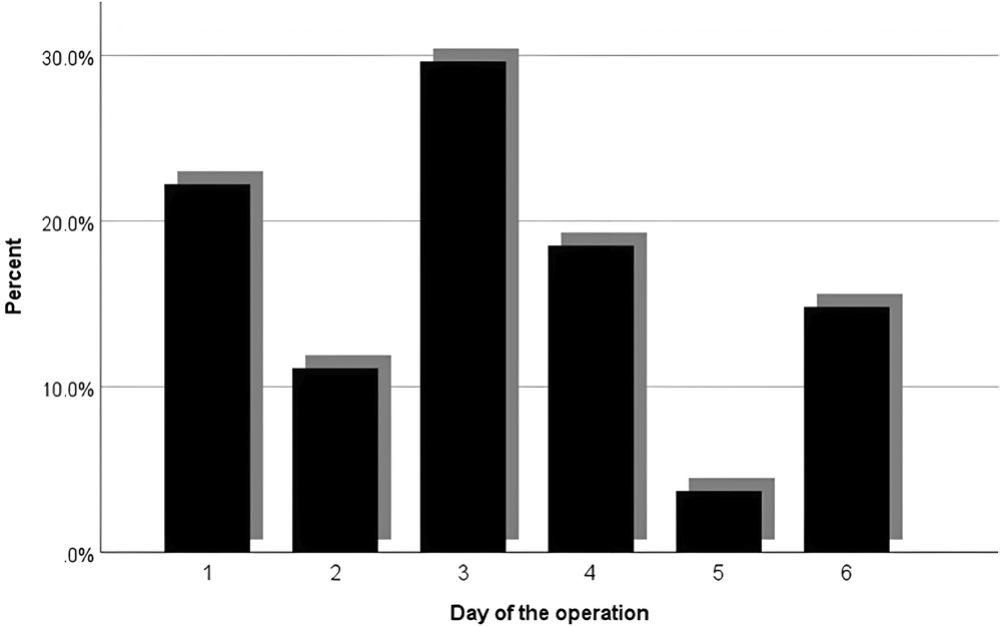
The conversion rate for each operating day.

**FIGURE 4:**
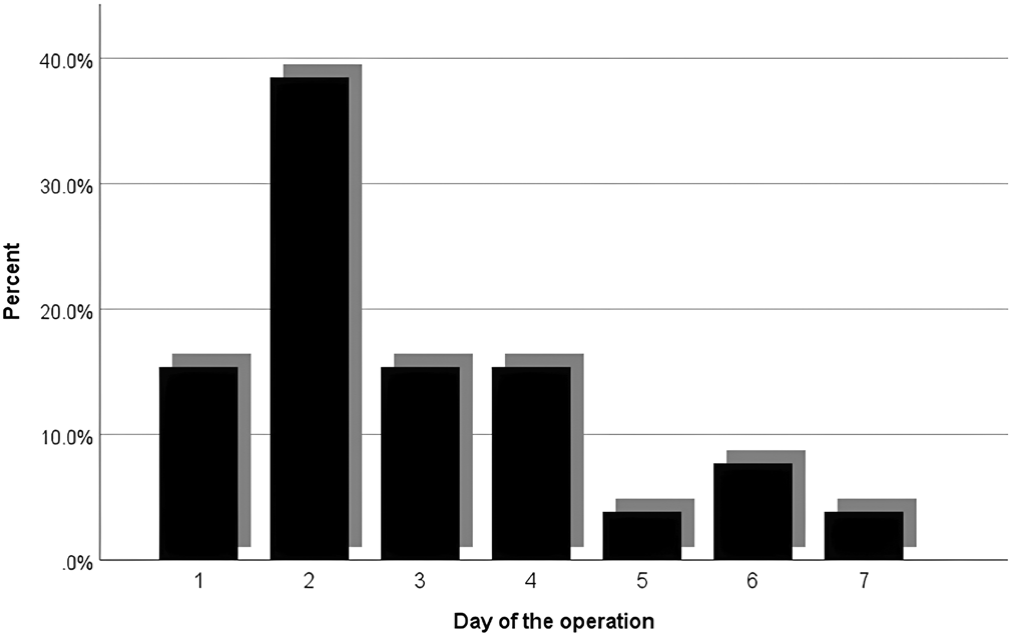
The subtotal cholecystectomy rate for each operating day.

The conversion from laparoscopic to open surgery occurred in 27 patients (0.8%) because of unclear anatomy resulting from severe inflammatory adhesion, intraoperative bleeding, common bile duct, and other adjacent organ injuries. There was a statistically significant difference for the patients who converted to open surgery, as 62.9% of the conversions happened in the first 3 days, as shown in Figure 3 (*p* = 0.011). In contrast, Figure 4 shows the rate of laparoscopic subtotal cholecystectomy in our study was 0.75%.

**FIGURE 5:**
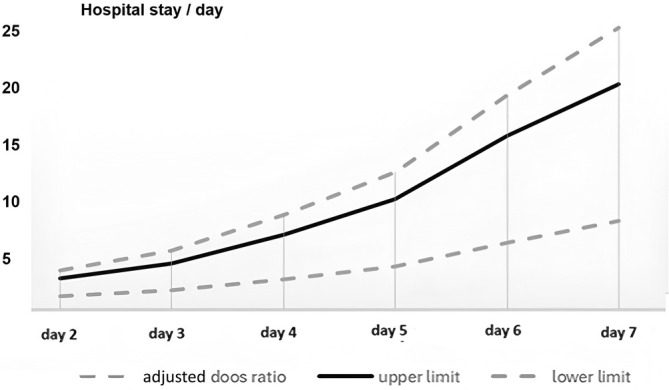
Changing the hospital stay according to the timing of surgery.

The overall reoperation rate was 0.5% (17 patients). It included three cases of common bile duct injury, three cases of bile leak, four cases of postoperative bleeding, three cases of gall bladder bed hematoma, two cases of small bowel injury, and two cases of wound collection. There were no significant changes in reoperation rates for the patients who underwent laparoscopic cholecystectomy on the day of admission or for those who were delayed until Day 7 (*p* = 0.831). Although Figure 5 shows a significantly longer hospital stay resulted from delaying laparoscopic cholecystectomy: this rose from slightly over 3 days for patients who had surgery on the day of admission to more days for patients who had surgery on Day 7 or later (median = 3, range from 1 to 197 days, *p* < 0.001).

Table S2 presents a comprehensive comparison of the crude and adjusted odds ratios for the postoperative outcomes grouped by operation day, whereas Table S3 represents a multivariable analysis to adjust for confounding variables that affect the outcomes of cholecystectomy after acute cholecystitis.

## 4 Discussion

The surgical treatment of acute cholecystitis could be time‐critical, where the timing of surgery in acute cholecystitis has historically been controversial because of the significant change in the complications rate. Early cholecystectomy is defined as the operation performed within the first 7 days of symptom onset, and delayed cholecystectomy is defined as that performed 6 weeks after the initial diagnosis [16]. Most international literature has concluded that early laparoscopic cholecystectomy has better outcomes and a lower cost [13, 17, 18]. Zhu et al. concluded that the inflammatory change in acute cholecystitis may not involve the Calot triangle area in the first 72 h following the onset of symptoms. Therefore, surgical dissection performed during this time seems safer and easier than surgery performed later; this is reflected in the low rates of complications and conversion to open surgery [19]. Other studies also concluded that the operation is still safe even after 72 h from symptom onset [20]. Therefore, it is suggested that early laparoscopic cholecystectomy may be offered to patients with acute cholecystitis within 7 days of the onset of symptoms.

The intraoperative timing of cholecystectomy refers to the duration of various stages involved in the surgical procedure. Furthermore, the intraoperative timing can vary depending on factors such as the surgical approach and the case's complexity. The mean operative time for laparoscopic cholecystectomy is typically less than 1 h [21]. The cumulative risk of intraoperative complications, such as four times for laparoscopic cholecystectomy lasting for 2 hs or more, regardless of the surgeon's skills [22]. Our study has shown that the average cholecystectomy lasts about 31–90 min. We had outlier results greater than the average. However, this could be explained because of possible complications arising, such as the common bile duct injury, which demands the involvement of the hepatobiliary surgeon, thus requiring more time. The stratification of days in all periods showed us that there was a significant difference in intraoperative duration between all groups from the first to the seventh day, with the variation beginning on the fourth day (*p* < 0.001). This is further highlighted by another study, which showed that intraoperative time was compared between a groups that had a laparoscopic cholecystectomy done within 24 h of the onset of symptoms versus another group that had an operation within 25–72 h of the onset of symptoms. It was found that there was no significant difference in the duration of the operation between both groups, with the median duration of surgery in the former being 70 min (IQR = 35) in comparison to the latter being 65 min (IQR = 30) with a *p* value of 0.23 [23]. Looking into studies that explored comparisons further than a week yielded a study that states intraoperative time was similar in patients undergoing laparoscopic cholecystectomy performed during the admission day versus patients that had the operation performed at least 6 weeks following the initial diagnosis (91 vs. 88 min; *p* < 0.910) [20].

Laparoscopic cholecystectomy complications have, according to a recent systematic review, a pooled prevalence range of 1.6%–5.3% [24]. These could range from bleeding, bile leaks, common bile duct injuries, and postoperative abscess formation. In our study, there was no significant difference between the complication rate and the time of operation every day during the first week (*p* = 0.373). Our findings are supported by another study that showed postoperative complications were similar in patients undergoing laparoscopic cholecystectomy performed during the admission day and patients that had operations performed at least 6 weeks following the initial diagnosis (15% vs. 17%; *p* = 1.000), respectively [20]. Despite what is commonly known, the rate of complications would increase with the delay of the operation. These findings could be explained by early detection, a low diagnostic threshold, the early initiation of antibiotics, and the improvement of surgical techniques.

Intraoperative difficulty in laparoscopic cholecystectomy refers to the level of surgical challenges encountered during the surgical procedure. Numerous scoring systems are attempting to quantify the difficulty preoperatively and intraoperatively, to varying degrees of success. One example developed from a systematic review of many studies uses gallbladder appearance and adhesions, as well as the degree of distention, the ease of access affected by body mass index or previous abdominal surgeries, local and septic complications due to leakage of pus or bile, and lastly, the identification of the cystic artery and duct, which can be altered because of anatomical variations and adhesions [25]. A study has demonstrated that increased intraoperative difficulty has led to an increased rate of conversion and subtotal cholecystectomy [26]. The rate of conversion from laparoscopic to open surgery in our study was 0.8%. That was because of unclear anatomy resulting from severe inflammatory adhesion, intraoperative bleeding, common bile duct, and other adjacent organ injuries. The reason for our low conversion rates (0.8%) is that surgeons in our institution tend to do laparoscopic subtotal cholecystectomy in challenging operations because of severe inflammatory changes and massive adhesions that cause anatomical landmarks to vanish, instead of converting the surgery to open. Our study has shown that early laparoscopic cholecystectomy has a significant difference in conversion rate compared with delayed laparoscopic cholecystectomy (*p* = 0.011), as 62.96% of cases happened in the first 3 days. Other studies suggest that the rate of conversion is reduced in the early laparoscopic cholecystectomy group (0–3 days: 3.6%; 4–7 days: 4.0%; ≥ 8 days: 4.7% [*p* < 0.001]) in comparison to the other study groups (4–7) and (8 and above) [27].

In our study, patients undergoing laparoscopic cholecystectomy have seen a median postoperative hospital stay of 4.22 days (range: 1–59). The preoperative waiting time was not included as there are cases that get delayed because of circumstances of high demand in the hospital and overcrowded lists, which resulted in bloated waiting times and would cause bias. Reduction of hospital stays has been shown to reduce hospital complications (odds ratio: 0.58; 95% confidence interval: 0.36–0.94) as well as a decrease in cost [28]. The median hospital stays difference was found to be insignificant in the group that had laparoscopic cholecystectomy done within 24 h of symptom onset versus the other group that had the operation within 25–72 h of symptom onset (*p* value 0.65) [23]. In our study, significantly longer hospital stays resulted from delaying laparoscopic cholecystectomy; this rose from slightly over 3 days for patients who had surgery on the day of admission to more days for patients who had surgery on day four or later, as shown in Figure 5 (median = 3, range from 1 to 197 days, *p* < 0.001). Like that, the mean duration of hospital stays in the group that had their laparoscopic cholecystectomy done within 72 h of admission was 1.67 days as compared with 1.47 days in the delayed group that had their operation performed after 6–8 weeks, with the difference being statistically insignificant (*p* value 0.379) [29]. The length of hospital stay was compared in a study that housed three groups. The first group was patients who received cholecystectomy within 3 days of admission; the second group was patients who underwent cholecystectomy between days four and seven following admissions, and the third group was patients who received cholecystectomy 8 days following their admission. It was found that early cholecystectomy within 3 days of admission was also associated with a shorter postoperative hospital stay (*p* < 0.001) [27].

Total mean hospital costs in patients undergoing laparoscopic cholecystectomy performed early on symptoms onset day, versus patients that had laparoscopic cholecystectomy performed at least 6 weeks following the initial diagnosis, were 9349 euros and 12 361 euros, respectively. The mean difference in hospital costs was 3012 euros per patient in favor of early laparoscopic cholecystectomy (*p* < 0.018) [19].

According to our results, we can see that the acute cholecystitis patient may have longer operative duration and hospital stays if surgery scheduling is delayed beyond 3 days after the onset of pain. This could be explained by the pathophysiology of acute cholecystitis and how the inflammatory process changes over time, wheres during the phase of edematous cholecystitis, which is characterized by hyperemic thick tissue phlegmon change that eventually progresses with the passage of days to purulent and necrotizing change if not controlled by the proper therapeutic approach [30]. All this local inflammation exacerbation makes it more difficult to dissect tissues and recognize structures during the surgery, which results in a longer operative duration and a hospital stay for additional intravenous antibiotic treatment. In conclusion, our study shows delays in laparoscopic cholecystectomy scheduling for acute cholecystitis after 3 days from the onset of symptoms can lead to a longer operative duration as well as longer hospital stays. However, it doesn't significantly impact overall complications and reoperation rates, allowing a feasible and safe procedure to be performed within 7 days.

## Ethics Statement

The study was approved by the ethics committee (IRB number: MRC‐01‐24‐236).

## Conflicts of Interest

The authors declare no conflicts of interest.

## Supporting information


**Table S1.** Postoperative complication of study population.
**Table S2.** Comparison of crude and adjusted odds for the outcomes. All the results are relative to time of operation.
**Table S3.** Factors affect the outcomes of cholecystectomy after acute cholecystitis.

## Data Availability

The data that support the findings of this study are available on request from the corresponding author. The data are not publicly available due to privacy or ethical restrictions.
