# Supplementary material for: Early Laparoscopic Cholecystectomy for Acute Cholecystitis. When Do Risks Seem Imminent?
Source: Asian J Endosc Surg. 2025 May 6;18(1):e70052. doi: 10.1111/ases.70052 (PMC12055317; doi:10.1111/ases.70052)
Supplement: Supplementary file 1 — Table S1. Postoperative complication of study population. Table S2. Comparison of crude and adjusted odds for the outcomes. All the results are relative to time of operation. Table S3. Factors affect the outcomes of cholecystectomy after acute cholecystitis. [file ASES-18-e70052-s001.docx]

**Early Laparoscopic Cholecystectomy for Acute Cholecystitis. When do Risks Seem Imminent?**

**Corresponding Author**

Dr. Salah Mansor. Associate Professor & Acute Care Surgeon

Acute Care Surgery Section, Hamad General Hospital, HMC, Doha, Qatar.

General, Trauma and Acute Care Surgery Department, Al-Jalla Teaching Hospital, Benghazi University, Benghazi, Libya.

Phone: 00974 55633006

Email: [Smansor@hamad.qa](mailto:Smansor@hamad.qa%20y)

ORCID ID / 0000-0001-9878-4277

| No | Complication | No | % |
| --- | --- | --- | --- |
| 1 | Common Bile Duct injury | 8 | 0.25% |
| 2 | Postoperative elevated Liver Function Test | 42 | 1.27% |
| 3 | Bile leak | 21 | 0.63% |
| 4 | Gall bladder bed bleeding | 19 | 0.57% |
| 5 | Postoperative retained CBD stone | 19 | 0.57% |
| 6 | Iatrogenic gall bladder injury | 17 | 0.51% |
| 7 | Gall bladder bed collection | 15 | 0.45% |
| 8 | Postoperative pancreatitis | 14 | 0.42% |
| 9 | Gall bladder bed hematoma | 7 | 0.21% |
| 10 | Cystic artery bleeding | 6 | 0.18% |
| 11 | Iatrogenic cystic duct injury | 3 | 0.09% |
| 12 | Biliary peritonitis | 1 | 0.03% |
| 13 | liver parenchyma injury | 13 | 0.39% |
| 14 | Duodenal injury | 3 | 0.09% |
| 15 | Small bowel injury | 3 | 0.09% |
| 16 | Gastric injury | 1 | 0.03% |
| 17 | Serosal tear in transverse colon | 1 | 0.03% |
| 18 | Portal vein injury | 1 | 0.03% |
| 19 | Mesenteric hematoma by Veress needle | 1 | 0.03% |
| 20 | Port site bleeding | 8 | 0.24% |
| 21 | Postoperative bleeding | 2 | 0.06% |
| 22 | Wound infection | 19 | 0.57% |
| 23 | Wound hematoma | 8 | 0.24% |
| 24 | Wound Seroma | 1 | 0.03% |
| 25 | Surgical emphysema | 4 | 0.12% |
| 26 | Sepsis | 5 | 0.15% |
| 27 | Atelectasis | 2 | 0.06% |
| 28 | Incisional hernia | 10 | 0.30% |
| 29 | Myocardial infarction | 1 | 0.03% |
| 30 | Pneumothorax | 1 | 0.03% |
| 31 | Postoperative Ileus | 1 | 0.03% |
| 32 | Pleural effusion | 1 | 0.03% |
| 33 | Pulmonary edema | 1 | 0.03% |
| 34 | Pulmonary embolism | 1 | 0.03% |
| 35 | Urinary Retention | 1 | 0.03% |

Table 3. Postoperative complication of study population.

| **Variable** | **Crude Odds ratio** | **Cl 95%** | **P value** | **Adjusted odds ratio** | **Cl 95%** | **P value** |
| --- | --- | --- | --- | --- | --- | --- |
| **Operative Duration**  Day 1  Day 2  Day 3  Day 4  Day 5  Day 6  Day 7 | 1.097  1.252  1.989  2.109  2.288  1.683 | (0.825-1.457)  (0.920-1.704)  (1.430-2.766)  (1.407-3.161)  (1.455-3.6)  (1.096-2.584) | .  0.525  0.153  0.001  0.001  0.001  0.017 | 0.937  1.492  2.232  2.853  4.555  6.797 | (0.599-1.466)  )0.946-2.355)  (1.387-3.592)  (1.659-4.905)  (2.6-7.979)  (4.125-11.19) | 0.775  0.085  0.001  <0.001  <0.001  <0.001 |
| **Total Complication**  Day 1  Day 2  Day 3  Day 4  Day 5  Day 6  Day 7 | 1.073  1.519  1.386  1.189  1.277  0.909 | (0.703-1.638)  (0.980-2.353)  (0.840-2.287)  (0.622-2.272)  (0.613-2.663)  (0.439-1.884) | 0.743  0.062  0.202  0.601  0.514  0.798 | 1.078  1.517  1.346  1.125  1.205  0.97 | (0.705-1.65)  (0.976-2.358)  (0.813-2.229)  (0.585-2.164)  (0.574-2.531)  (0.467-2.018) | 0.729  0.064  0.249  0.723  0.622  0.936 |
| **Conversion Rate**  Day 1  Day 2  Day 3  Day 4  Day 5  Day 6  Day 7 | 0.289  1.177  1.449  -  0.916  - | (0.064-1.299)  (0.342-4.054)  (0.386-5.45)  -  (0.098-8.047)  - | 0.106  0.796  0.583  0.996  0.889  0.996 | 0.288  1.174  1.450  -  0.810  - | (0.064-1.298)  (0.340-4.054)  (0.383-5.484)  -  (0.089-7.401)  - | 0.105  0.799  0.584  0.996  0.852  0.996 |
| **Re-Operation**  Day 1  Day 2  Day 3  Day 4  Day 5  Day 6  Day 7 | 1.098  1.278  1.357  -  1.385  - | (0.283-4.261)  (0.157-3.893)  (0.272-6.757)  -  (0.143-13.41)  - | 0.893  0.765  0.71  0.996  0.779  0.996 | 1.108  0.779  1.294  -  1.279  - | (0.284-4.317)  (0.156-3.884)  (0.259-6.47)  -  (0.131-12.51)  - | 0.883  0.761  0.754  0.995  0.832  0.996 |
| **Hospital Stay**  Day 1  Day 2  Day 3  Day 4  Day 5  Day 6  Day 7 | 0.936  1.404  2.300  3.298  5.041  5.815 | (0.644- 1.36)  (0.955- 2.064)  (1.541- 3.433)  (2.1- 5.18)  (3.123- 8.13)  (3.771- 8.96) | 0.729  0.085  <0.001  <0.001  <0.001  <0.001 | 0.933  1.389  2.249  3.219  5.002  6.678 | (0.639- 1.363)  (0.939- 2.055)  (1.496- 3.382)  (2.025- 5.115)  (3.059- 8.181)  (4.287- 10.4) | 0.721  0.10  <0.001  <0.001  <0.001  <0.001 |

Table 4. Comparison of crude and adjusted odds for the Outcomes. All the Results are Relative to time of Operation.

| **Variable** | **Crude Odds ratio** | **Cl 95%** | **P value** | **Adjusted odds ratio** | **Cl 95%** | **P value** |
| --- | --- | --- | --- | --- | --- | --- |
| Complication  Age  Gender  Diabetes mellitus  Past abdominal surgery | 0.477  1.974  1.843  1.035 | (0.311-0.732)  (1.496-2.603)  (1.34-2.523)  (0.757-1.41) | 0.001  <0.001  <0.001  0.830 | 0.578  2.017  1.597  1.279 | (0.368-0.909)  (1.514-2.686)  (1.147-2.223)  (0.923-1.772) | 0.018  <0.001  0.006  0.140 |
| Intra operative complications  Age  Gender  Diabetes mellitus  Past abdominal surgery | 0.425  2.323  1.606  0.820 | (0.238-0.759)  (1.534- 3.519)  (1.01-2.554)  (0.505-1.329) | 0.004  <0.001  0.045  0.420 | 0.493  2.288  1.323  1.056 | (0.266-0.912)  (1.492-3.507)  (0.809-2.164)  (0.641-1.740) | 0.024  <0.001  0.264  0.831 |
| Post operative complication  Age  Gender  Diabetes mellitus  Past abdominal surgery | 1.624  1.843  2.017  1.08 | (0.97-2.7)  (1.31-2.58)  (1.38-2.93)  (0.74-1.58) | 0.062  <0.001  <0.001  0.675 | 0.834  1.888  1.879  1.290 | (0.460-1.514)  (1.332-2.676)  (1.273-2.774)  (0.870-1.913) | 0.552  <0.001  0. 002  0.205 |
| Reoperation  Age  Gender  Diabetes mellitus  Past abdominal surgery | 0.492  0.039  2.313  2.577 | (0.112-2.16)  (1.05-10)  (0.811-6.59)  (0.736-9.025) | 0.348  3.254  0.117  0.139 | 0.678  3.384  1.995  1.459 | (0.142-3.240)  (1.066-10.743)  (0.662-6.011)  (0.458-4.649) | 0.627  0.039  0.220  0.523 |
| Biliary complication  Age  Gender  Diabetes mellitus  Past abdominal surgery | 2.713  0.467  0.509  1.127 | (1.71-4.28)  (0.336-0.649)  (0.355-0.729)  (0.77-1.65) | <0.001  <0.001  <0.001  0.538 | 2.203  0.473  0.623  0.891 | (1.354-3.585)  (0.336-0.664)  (0.425-0.912)  (0.599-1.323) | 0.001  0.001  0.015  0.566 |
| Conversion  Age  Gender  Diabetes mellitus  Past abdominal surgery | 0.822  2.868  1.945  1.443 | (0.193-3.497)  (1.209-6.8)  (0.818-4.62)  (0.629-3.31) | 0.791  0.017  0.132  0.386 | 1.056  3.261  1.810  1.956 | (0.235-4.74)  (1.336-7.96)  (0.737-4.44)  (0.827-4.62) | 0.943  0.009  0.196  0.127 |
| Post operative hospital stay  Age  Gender  Diabetes mellitus  Past abdominal surgery | 0.336  1.981  2.164  0.743 | (0.235-0.48)  (1.550-2.532)  (1.64-2.84)  (0.549-1.004) | <0.001  <0.001  <0.001  0.053 | 0.430  1.880  1.786  0.906 | (0.294-0.628)  (1.459-2.422)  (1.336-2.389)  (0.662-1.240) | <0.001  <0.001  <0.001  0.538 |
| Operation duration  Age  Gender  Diabetes mellitus  Past abdominal surgery | 0.308  1.776  1.508  0.884 | (0.203-0.467)  (1.360-2.319)  (1.098-2.071)  (0.646-1.21) | <0.001  <0.001  0.011  0.443 | 0.342  1.745  1.166  1.088 | (0.219-0.535)  (1.322-2.303)  (0.828-1.643)  (0.785-1.509) | <0.001  <0.001  0.379  0.612 |

Table 5. Factors affect the outcomes of cholecystectomy after acute cholecystitis,

Analyzing other factors that may affect the outcomes of the cholecystectomy after acute cholecystitis, regardless of whether the patient was operated on immediately on the first or delayed to the seventh day, it was found that complications increase by 2.017 if the patient was female, and the risk of complications also increases by 1.597 if she has diabetes. In both cases, the statistical analysis takes into account the different ages of patients and also that they may have a history of abdominal surgery. The length of the operation may increase in a magnitude of 1.745 if the patient is female. The probability of reoperation increases by 3.384 if the patient is female.

The risk of developing biliary complications increases if the patient is over 65 years old. The chance of converting the operation to an open surgery increases by 3.261 if the patient is female. The chance of elongated post-operative hospital stay increases by 1.8 if the patient is a female with diabetes.
